# Complete Genome Sequence of Bacteriocin-Producing Enterococcus faecium HY07

**DOI:** 10.1128/MRA.00276-19

**Published:** 2019-09-19

**Authors:** Xiyu Duan, Hui Yang, Yun Tian, Rongrong Wang, Chengguo Liu, Hui Zhou

**Affiliations:** a College of Bioscience and Biotechnology, Hunan Agricultural University, Changsha, China; b Hunan Province University Key Laboratory for Agricultural Biochemistry and Biotransformation, Hunan Agricultural University, Changsha, China; c Hunan Co-Innovation Center for Utilization of Botanical Functional Ingredients, Changsha, China; d College of Food Science and Technology, Hunan Agricultural University, Changsha, China; University of Maryland School of Medicine

## Abstract

Here, we report the draft genome sequence of the bacteriocin-producing Enterococcus faecium strain HY07, isolated from traditional Chinese fermented sausages. The genome comprises 2,585,631 bp with 2,624 coding sequences, as assigned by NCBI, which may provide fundamental molecular information on elucidating the adaption mechanism of Enterococcus faecium to the meat environment.

## ANNOUNCEMENT

Enterococci are found in many food products, especially those of animal origin, seafood, and dairy products. Enterococci are the most thermotolerant nonsporulating bacteria, and some can even survive pasteurization temperatures. Tolerance to environmental extremes explains their survival during the processing of cooked and uncooked cured meats, as well as their ability to multiply during fermentations (1). On the one hand, the species Enterococcus faecium was considered a harmless commensal of the mammalian gastrointestinal (GI) tract and is commonly used as a probiotic. On the other hand, some of the E. faecium strains are typical opportunistic pathogens that cause diseases, especially in nosocomial settings, which may, in part, be linked to the presence of virulence determinants and antibiotic resistance (2).

In this study, we report on the genome sequence of E. faecium HY07, which was isolated from traditional Chinese fermented sausage. We also found that E. faecium HY07 exhibited antimicrobial activity against Listeria, Salmonella, Shigella, Staphylococcus, Clostridium, and Bacillus species (3).

E. faecium HY07 was anaerobically cultured overnight at 37°C in de Man, Rogosa, and Sharpe (MRS) broth. Genomic DNA was extracted using phenol-chloroform extraction with bead beating, and the genomic libraries were prepared using a sequencing kit (SQK-MAP-006; Oxford Nanopore Technologies, Oxford, UK) according to the manufacturer’s instructions. Pacific Biosciences sequencing technology was then used to sequence the genomic DNA isolated from E. faecium HY07. Trimmomatic version 0.38 was used to trim low-quality reads. A total of 98,024 reads were generated at an average read length of 9,193 bp, with an average depth of coverage of 432.70-fold. Corrected reads were assembled using the Celera Assembler (4). Protein-coding sequences were predicted by GLIMMER software version 3.02 (5) and annotated using BLAST searches of nonredundant (Nr) protein sequences from the NCBI, clusters of orthologous groups (COG), Gene Ontology (GO), and Kyoto Encyclopedia of Genes and Genomes (KEGG) databases (6–8). tRNA genes were detected using tRNAscan-SE 1.23, and rRNA genes were detected using RNAmmer 1.2 (9, 10).

The complete genome of E. faecium HY07 is composed of a 2,585,631-bp chromosome with a G+C content of 38.34% and two plasmids, namely, a plasmid of 258,852 bp with a G+C content of 36.18% and another plasmid of 21,516 bp with a G+C content of 33.55%. Using GLIMMER, 2,838 open reading frames (ORFs) were predicted in E. faecium HY07, including 2,624 protein-coding genes, 67 tRNA-coding genes, and 18 rRNA-coding genes.

Several coding DNA sequences (CDSs) for the production of bacteriocins, namely, enterocin P (GenBank accession number AYA33186), which contains the consensus class IIa bacteriocin motif YGNGV in the *N*-terminal region, were found (11). The genes encoding bacteriocins (GenBank accession numbers AYA33274, AYA35563, and AYA35573) were found in the genome of E. faecium HY07.

A search for virulence factors associated with invasiveness and disease severity was conducted (12). The genes involved in the resistance to antimicrobials, such as copper, penicillin, tetronasin, and vancomycin, were found in the genome of E. faecium HY07.

These data provide insights into the genetic basis of bacteriocin-producing E. faecium HY07 in relation to the meat environment.

### Data availability.

The complete genome sequence project has been deposited in GenBank under the accession numbers CP032307 (plasmid), CP032308 (chromosome), and CP032309 (plasmid). The versions described in this paper are the first versions, CP032307.1, CP032308.1, and CP032309.1. Raw sequencing reads are available in NCBI BioProject under the accession number PRJNA416245.
